# Grape seed proanthocyanidins prevent irradiation-induced differentiation of human lung fibroblasts by ameliorating mitochondrial dysfunction

**DOI:** 10.1038/s41598-017-00108-9

**Published:** 2017-03-03

**Authors:** XiaoHong Yang, Tao Liu, Bo Chen, Fangqin Wang, Qunfang Yang, XiaoHong Chen

**Affiliations:** Department of Pharmacology, College of Pharmacy, Third Military Medical University, Chongqing, 400038 China

## Abstract

Radiation-induced lung fibrosis (RILF) is a long-term adverse effect of curative radiotherapy. The accumulation of myofibroblasts in fibroblastic foci is a pivotal feature of RILF. In the study, we found the inhibitory effect of grape seed proanthocyanidins (GSPs) on irradiation-induced differentiation of human fetal lung fibroblasts (HFL1). To explore the mechanism by which GSPs inhibit fibroblast differentiation, we measured the reactive oxygen species (ROS) levels, mitochondrial function, mitochondrial dynamics, glycolysis and the signaling molecules involved in fibroblast transdifferentiation. GSPs significantly reduced the production of cellular and mitochondrial ROS after radiation. The increases in mitochondrial respiration, proton leak, mitochondrial ATP production, lactate release and glucose consumption that occurred in response to irradiation were ameliorated by GSPs. Furthermore, GSPs increased the activity of complex I and improved the mitochondrial dynamics, which were disturbed by irradiation. In addition, the elevation of phosphorylation of p38MAPK and Akt, and Nox4 expression induced by irradiation were attenuated by GSPs. Blocking Nox4 attenuated irradiation-mediated fibroblast differentiation. Taken together, these results indicate that GSPs have the ability to inhibit irradiation-induced fibroblast-to-myofibroblast differentiation by ameliorating mitochondrial dynamics and mitochondrial complex I activity, regulating mitochondrial ROS production, ATP production, lactate release, glucose consumption and thereby inhibiting p38MAPK-Akt-Nox4 pathway.

## Introduction

Radiation therapy is an effective treatment for multiple thoracic malignancies. However, the severe side effects of radiotherapy, such as pneumonitis and lung fibrosis, which are generally called radiation-induced lung fibrosis (RILF), compromise the curative rate and wellbeing of lung cancer patients. The incidence of RILF varies from 1% to 43%^1^. Understanding of the detailed mechanisms of RILF is urgently needed for the development of therapies to reduce or control RILF.

Although the exact mechanisms of RILF are unclear, accumulating evidence suggests that it involves increased deposition and altered composition of extracellular matrix (ECM) during chronic injury. Furthermore, the appearance of fibroblastic foci is a pathological hallmark of fibrosis^2–4^. Fibroblasts are the predominant secretory cells of ECM proteins in the lung and are also key mediators of normal and pathological lung remodeling. During pulmonary fibrogenesis, fibroblasts proliferate and differentiate into myofibroblasts, which function as key “effector” cells and are characterized by α-smooth muscle actin (α-SMA) expression and increased generation and secretion of ECM proteins (i.e., collagen and fibronectin) that contribute to fibrotic disorders^5–7^. Therefore, the myofibroblasts that accumulate in fibroblastic foci are regarded as the pivotal promoters of pulmonary fibrosis, and inhibition of the fibroblast-to-myofibroblast differentiation process may reveal additional targets for lung fibrosis treatment.

Myofibroblast differentiation in response to irradiation is triggered by many factors such as cytokines, growth factors and components of the ECM^8^. Of the key elements of fibrogenesis, reactive oxygen species (ROS) are closely linked to the differentiation of many types of cells, such as hepatic stellate cells and adventitial fibroblasts^9,10^. It has been well documented that irradiation produces sufficient ROS to drive myofibroblast differentiation in normal human lung fibroblasts. Mitochondria are the main site of intracellular oxygen consumption and the main source of ROS formation^11,12^. Therefore, it is possible that mitochondrial ROS generation is crucial for fibroblast differentiation after irradiation. Thus, improving mitochondria function might be a promising strategy for inhibiting fibroblast differentiation.

Grape seed proanthocyanidins (GSPs), a group of proanthocyanidins that primarily contain dimers, trimers and other oligomers of catechin and epicatechin and their gallic acid esters, have been reported to protect against oxidation injury and to scavenge oxygen free radicals^13–15^. A recent study showed that proanthocyanidins could ameliorate H_2_O_2_-induced mitochondrial dysfunction by promoting mitochondrial respiratory chain complex IV and reducing the levels of mitochondrial superoxide^16^. Moreover, grape seed extract can reduce silica-induced and bleomycin-induced pulmonary fibrosis in rats^17^, and GSPs protect mice from RILI by inhibiting the TGF-β1/Smad3/Snail signaling pathway, scavenging hydroxyl radicals (•OH) and modulating the levels of RILI-associated cytokine (interferon-γ, IL-4 and IL-13) derived from Th1/Th2 cells^18^. Taken together, these results show that GSPs have strong anti-oxidation, anti-fibrosis and anti-cancer effects against lung disease. However, whether GSPs ameliorate irradiation-induced pulmonary fibrosis by affecting the main source of ROS derived from the mitochondrial electron transport chain (ETC) has not yet been reported.

Therefore, our study was conducted to determine the inhibitory effect of GSPs on the differentiation of irradiated human fetal lung fibroblast cells (HFL1) and to explore the association between the repair of mitochondrial dysfunction triggered by irradiation and the antioxidant effects of GSPs. Our results suggest that GSPs prevent fibrosis by ameliorating the mitochondria fission/fusion imbalance and complex I activity in human lung fibroblasts and thereby ameliorating irradiation-induced mitochondrial dysfunction.

## Results

### GSPs inhibited irradiation-induced myofibroblast differentiation

**Figure 1:**
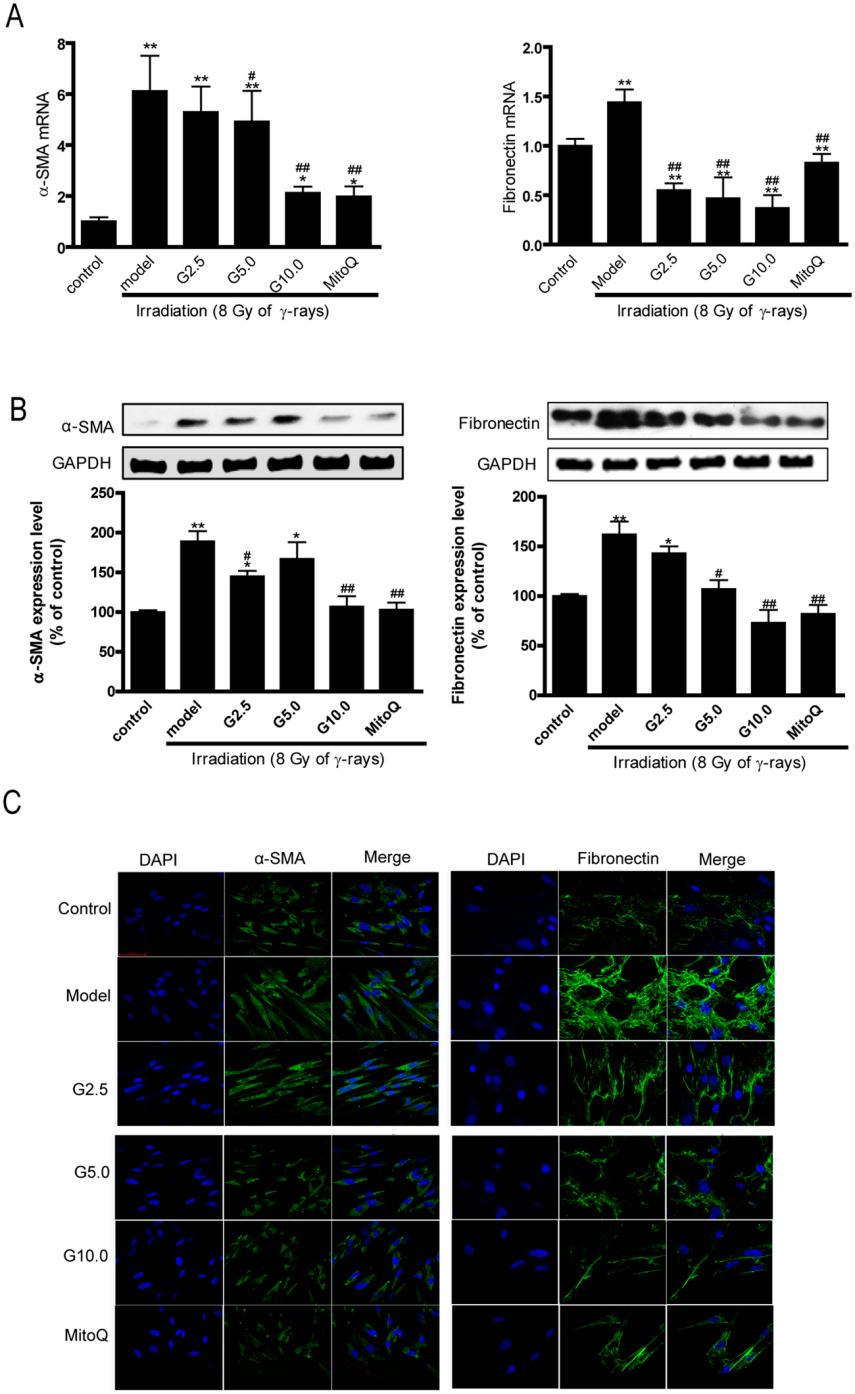
Expression of α-SMA and fibronectin in irradiation-induced HFL1 cells. HFL1 cells were pre-treated with GSPs (2.5, 5, 10 μg/ml) or MitoQ (200 nmol/L) for 24 hours before irradiation (γ-ray, 8 Gy) and were then cultured for another 72 h. The expression of the indicated genes was analyzed in the cells by qRT–PCR (**A**) and Western blotting (**B**). (**C**) Cells were immunostained for α-SMA and fibronectin. Confocal microscopy was performed. A representative image is shown from three replicates. Scale bars: 50 µM. Data are expressed as the mean ± SEM of three independent experiments. *P < 0.05 and **P < 0.01 versus control group. ^#^*P* < 0.05 and ^##^*P* < 0.01 versus model group. Abbreviations: G2.5, 2.5 μg/ml GSPs; G5, 0.5 μg/ml GSPs; G10, 10 μg/ml GSPs.

To investigate the effect of GSPs on irradiated HFL1 cells, we pre-treated HFL1 cells with GSPs (2.5, 5 or 10 μg/ml) or MitoQ (200 nmol/L) for 24 h before irradiation and examined the expression of α-SMA and fibronectin in HFL1 cells 72 h after irradiation by qRT-PCR, Western blot and immunofluorescent assays. As shown in Fig. 1A, after irradiation, the mRNA expression of *α-SMA* and *fibronectin* was significantly increased (*P* < 0.01) compared with the control HFL1 cells. When cells were pre-treated with GSPs (2.5, 5.0 or 10.0 μg/ml) for 24 h before irradiation, the expression of *α-SMA* and *fibronectin* was reduced (*P* < 0.01). To verify the effect of GSPs on the expression of α-SMA and fibronectin, we measured the expression levels of these two proteins by Western blot and immunofluorescent assays. As shown in Fig. 1B and C, GSPs also significantly inhibited α-SMA and fibronectin protein expression (*P* < 0.01), similar to the mRNA expression levels. Similar results were obtained using the mitochondria-targeted antioxidant MitoQ, which accumulates within mitochondria and is reduced to the antioxidant ubiquinol.

### GSPs decreased intracellular and mitochondrial ROS production in irradiated HFL1 cells

**Figure 2:**
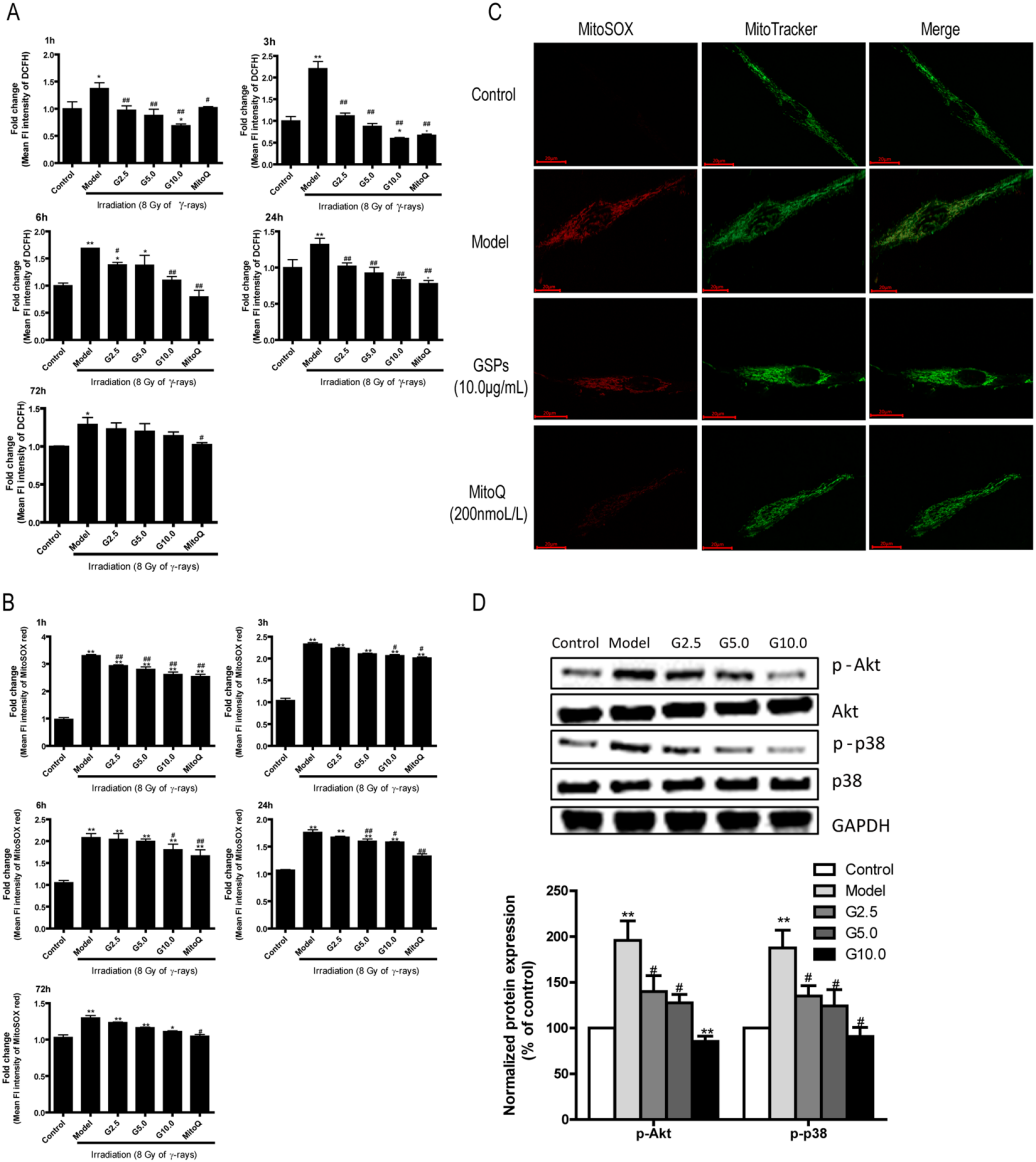
Effect of GSPs on intracellular and mitochondrial ROS production in irradiated HFL1 cells. (**A**) HFL1 cells were cultured for 1 h, 3 h, 6 h, 24 h or 72 h after irradiation with 8 Gy and incubated with 10 µM DCFH-DA for 15 min. (**B**) The above cells were treated with 5 µM MitoSOX red for 30 min. ROS levels were measured by flow cytometry. (**C**) A representative fluorescence microscope image of HFL1 cells co-stained with MitoSOX Red and MitoTracker at 72 h after 8 Gy of irradiation is shown. Scale bars: 20 µM. Red staining denotes MitoSOX-stained mitochondrial ROS. Green staining denotes MitoTracker-stained mitochondria. (**D**) GSPs decreased radiation-induced phosphorylation of p38MAPK and Akt during myofibroblast differentiation. After 8 Gy of irradiation, phosphorylation of p38MAPK and Akt was assessed 1 h after irradiation by Western blotting. Data are representative of at least three different experiments. Protein expression levels were normalized to that of GAPDH, and the fold increase is indicated under the relevant protein bands. *P < 0.05 and **P < 0.01 versus control group. ^#^*P* < 0.05 and ^##^*P* < 0.01 versus model group.

To determine whether the effect of GSPs inhibition on irradiation-induced HFL1 cell differentiation was associated with ROS production, we measured cellular ROS levels in irradiated cells pretreated with GSPs or MitoQ by flow cytometry using the fluorescent probe DCFDA. As shown in Fig. 2A, DCFDA fluorescence intensity in HFL1 cells gradually increased after irradiation, peaked at 3 h and declined at 6 h. Interestingly, when HFL1 cells were pretreated with GSPs for 24 h before γ-ray irradiation, the cellular ROS production was significantly reduced compared with the model group (*P* < 0.01). The mitochondria-targeted drug MitoQ had a similar effect to GSPs.

To determine whether irradiation-induced cellular ROS comes from mitochondria and GSPs affect mitochondrial ROS production in HFL1 cells, we analyzed mitochondrial superoxide generation production by flow cytometry and confocal microscopy using the fluorescent probe MitoSOX. After γ-ray irradiation, the MSR fluorescence intensity in cells peaked at 1 h and declined at 3 h, compared with the control group. Moreover, both GSPs (2.5, 5.0 or 10 μg/ml) and MitoQ (200 nm/L) suppressed irradiation-induced mitochondrial ROS production (Fig. 2B and C). MitoSOX is a reagent that enables us to specifically measure superoxide generation in the mitochondria of live cells. The specificity of mitochondrial ROS staining by MitoSOX was confirmed by double staining with MitoTracker. The MitoSOX signal was localized in MitoTracker-stained organelles, indicating that the MitoSOX staining was specific to mitochondria. The fluorescence assay also revealed that mitochondrial ROS increased markedly after irradiation, and pre-treatment with GSPs (10 μg/ml) or MitoQ (200 nm/L) significantly reduced the generation of mitochondrial ROS (Fig. 2C). To explore the signaling pathways involved in fibroblast transdifferentiation in response to mitochondrial ROS, the level of phosphorylation of p38MAPK and Akt were determined. As shown in Fig. 2D, phosphorylation of p38MAPK and Akt increased at 1 h after irradiation, and pretreatment with GSPs could decrease the level of phosphorylation of p38MAPK and Akt. In addition, the elevation of NADPH oxidase 4 (NOX4) expression induced by irradiation was attenuated by use of GSPs (Fig. S1A).

### The effect of GSPs on mitochondrial transmembrane potential in irradiated HFL1 cells

**Figure 3:**
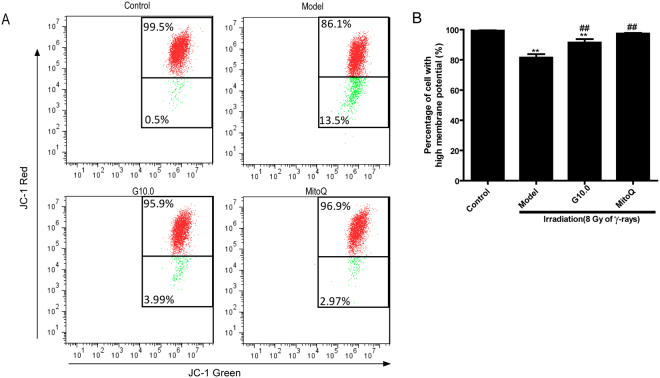
Effect of GSPs on mitochondrial membrane potential in irradiated HFL1 cells. (**A**) Flow cytometry analysis of JC-1 staining was used to assess mitochondrial membrane potential (MMP). HFL1 cells were pre-treated with GSP (10 μg/ml) or MitoQ (200 nmol/L) for 24 h before irradiation, and MMP was determined at 72 h after irradiation. One representative flow cytometry plot of the mitochondrial membrane potential is shown out of three replicates. The cells with enhanced red fluorescence had a higher membrane potential. The numbers in the corners represent the percentage of cells in the corresponding quadrants. (**B**) The percentage of cells with high MMP (%) is shown. Data are expressed as the mean ± SEM of three independent experiments. *P < 0.05 and **P < 0.01 versus control group. ^##^*P* < 0.01 versus model group.

To determine whether the effect of GSPs on mitochondrial ROS production was associated with changes in mitochondrial transmembrane potential, we next examined changes in irradiated cells pretreated with GSPs or MitoQ by flow cytometry using JC-1. As shown in Fig. 3A and B, irradiation led to a significant decrease in mitochondrial membrane potential compared to control cells, as indicated by the increase in green fluorescence. When cells were pre-treated with GSPs (10 µg/ml) or MitoQ (200 nm) for 24 h, the percentage of cells showing red fluorescence increased significantly (*P* < 0.01). These results imply that irradiation-induced ROS may be associated with a decrease in mitochondrial membrane potential (MMP), and that GSPs and MitoQ can ameliorate the reduction of MMP induced by irradiation.

### The effect of GSPs on mitochondrial dynamics in irradiated HFL1 cells

**Figure 4:**
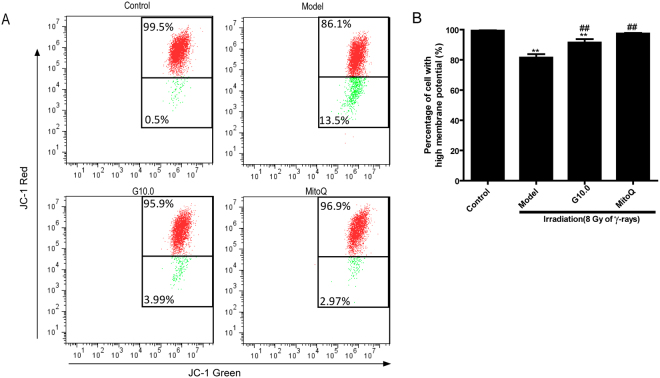
Effect of GSPs on mitochondrial dynamics in irradiated HFL1 cells. (**A**) Cells were prepared as described in the Methods and stained with MitoTracker® Green FM dye. Fluorescence images were collected by confocal microscopy. Scale bars: 20 µM. (**B**) Quantitative analysis of the percentage of fission cells is shown. (**C**) The mRNA levels of the mitochondrial fusion-related genes (mfn1, mfn2 and opa1) and fission-related genes (drp1 and fis1) were measured using qRT-PCR. Data are expressed as the mean ± SEM of three independent experiments. *P < 0.05 and **P < 0.01 versus control group. ^#^*P* < 0.05 and ^##^*P* < 0.01 versus model group.

Mitochondrial fission and fusion are known to play an important role in maintaining mitochondrial integrity and function. To characterize the effect of GSPs on changes in mitochondrial dynamics, we first monitored mitochondrial morphological changes by confocal microscopy. As shown in Fig. 4A and B, most of the control cells (>95%) showed normal, tubular mitochondria. However, after irradiation with 8 Gy, approximately 40% of cells contained normal tubular mitochondria like those seen in control cells, and >60% of irradiated HFL1 cells contained mitochondria with a fragmented, punctiform morphology. Moreover, pretreatment with GSPs or MitoQ protected mitochondria from fragmentation. We also measured the expression of mitochondrial fusion-related genes (*mfn1, mfn2* and *opa1*) and fission-related genes (*drp1* and *fis1*) by qRT-PCR. Figure 4C shows the effect of irradiation on the expression of the main fission- and fusion-related genes. Specifically, there was a statistically significant increase in *drp-1* expression and decrease in *mfn-1*/*2* expression after irradiation. GSPs or MitoQ pretreatment reduced the expression of *drp-1* and enhanced the expression of *mfn-1*/*2* (Fig. 4C).

### The effect of GSPs on ATP levels and glycolysis in irradiated HFL1 cells

**Figure 5:**
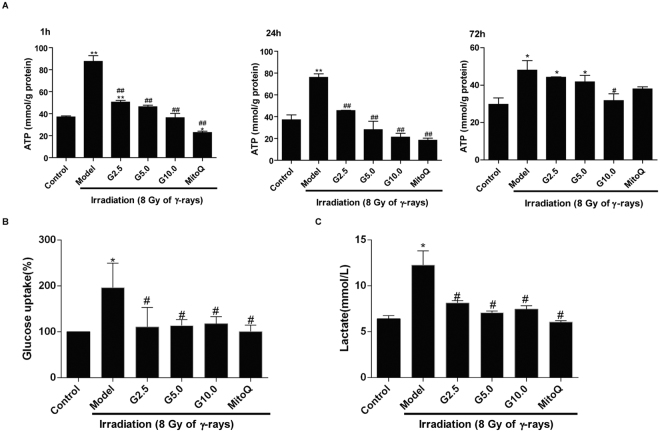
Treatment with GSP reduced the concentration of cellular ATP in irradiated HFL1 cells. HFL1 cells were pre-treated with GSP (2.5, 5, 10 μg/ml) or MitoQ (200 nmol/L) for 24 h before irradiation, and the cellular ATP content was determined at 1 h and 24 h after irradiation. Data are expressed as the mean ± SEM of three independent experiments. *P < 0.05 and **P < 0.01 versus control group. ^##^*P* < 0.01 versus model group.

Mitochondrial ATP content is a classic indicator of mitochondrial respiration. To investigate whether GSPs alter mitochondrial ATP production in irradiated HFL1 cells, the cellular ATP content of HFL1 cells was determined after treatment with GSPs. As shown in Fig. 5, ATP content was significantly increased in irradiated cells at 1 h, 24 h and 72 h (*P* < 0.01) after treatment with GSPs. Treatment with GSPs at doses of 2.5, 5.0 or 10 μg/ml significantly decreased ATP production in a dose-dependent manner (*P* < 0.01). MitoQ treatment also reduced ATP content at 1 h and 24 h.

Furthermore, to identify the effect of GSPs on glycometabolism, lactate release and glucose consumption were examined in irradiated HFL1 at 72 h. As shown in Fig. 5B, irradiation significantly increased glucose consumption and pretreatment with GSPs or MitoQ could attenuate glucose uptake in irradiated cells (*P* < 0.05). Concomitantly, lactate release of irradiated HFL1 was increased (*P* < 0.05), whereas pretreatment with GSPs attenuated the lactic acid accumulation caused by irradiation (*P* < 0.05) (Fig. 5C).

### The effect of GSPs on mitochondrial respiration in irradiated HFL1 cells

**Figure 6:**
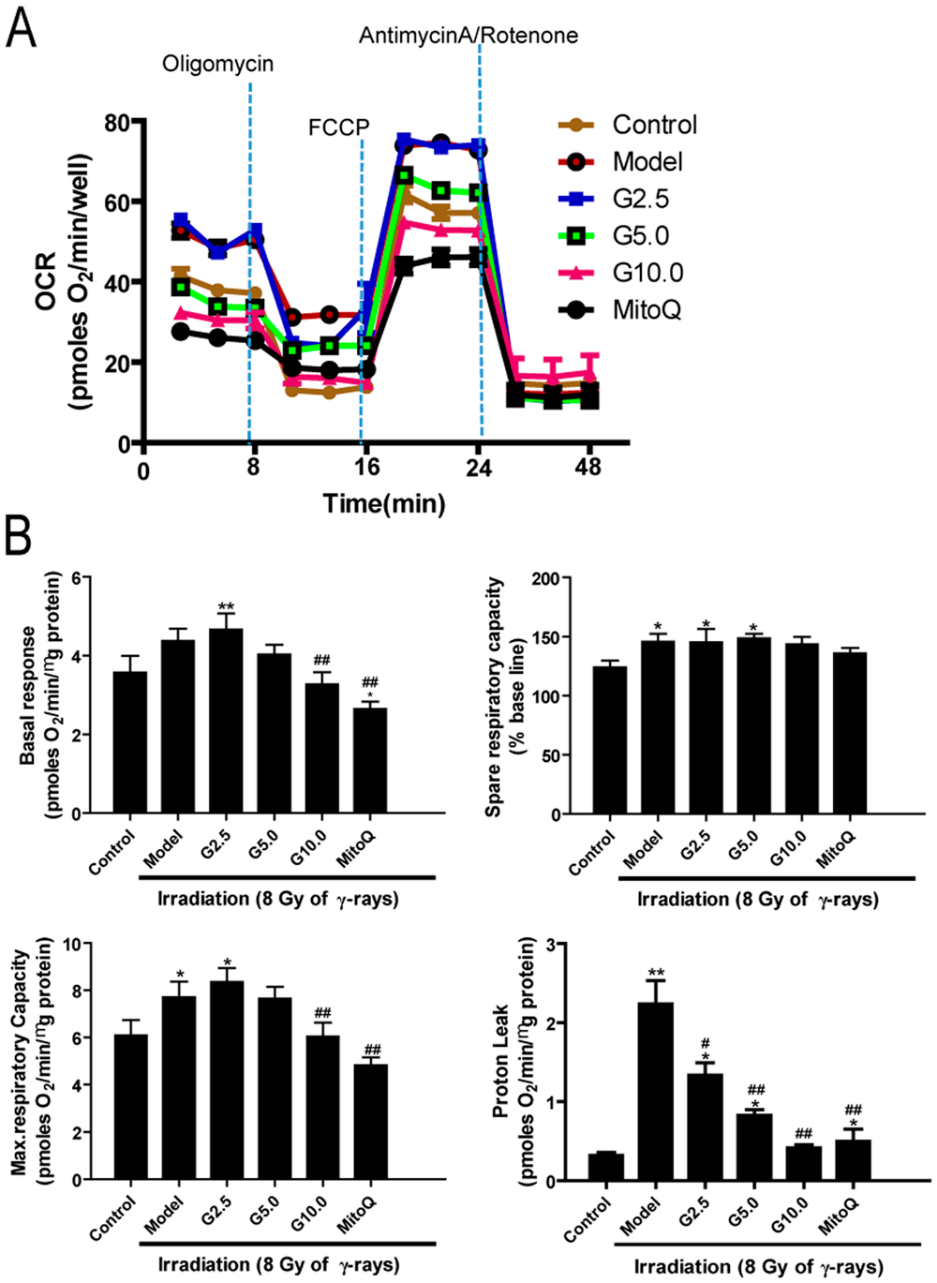
Effect of GSPs on mitochondrial respiration in irradiated HFL1 cells. (**A**) Oxygen consumption rates (OCR) in irradiated HFL1 cells exposed sequentially to oligomycin, FCCP and antimycin plus rotenone were measured 24 h after 8 Gy of irradiation. (**B**) The basal respiration, maximal respiratory capacity, spare respiratory capacity and proton leak of HFL1 cells were analyzed. Data are expressed as the mean ± SEM of three independent experiments. *P < 0.05 and **P < 0.01 versus control group. ^#^*P* < 0.05 and ^##^*P* < 0.01 versus model group.

To explore the possible mechanisms by which GSPs alter mitochondrial ROS production, we next tested whether irradiation affected cellular respiration. Cellular oxygen consumption (OCR) was monitored by XF96 using ETC inhibitors. As shown in Fig. 6A, irradiation significantly increased the rate of oxygen consumption compared with control cells. The presence of GSPs (10 μg/ml) or MitoQ inhibited the basal stimulation of OCR by irradiation. Furthermore, the maximal respiratory capacity induced by FCCP was approximately 1-fold higher in irradiated cells compared with control cells, and GSPs or MitoQ decreased the maximal respiratory capacity of irradiated cells to levels comparable with control cells (Fig. 6A and B). Similarly, irradiation increased the proton leak of HLF1 cells compared with control cells, and treatment with GSPs or MitoQ decreased the proton leak (*P* < 0.01).

### The effect of GSPs on mitochondrial respiratory chain complexes I and III in irradiated HFL1 cells

**Figure 7:**
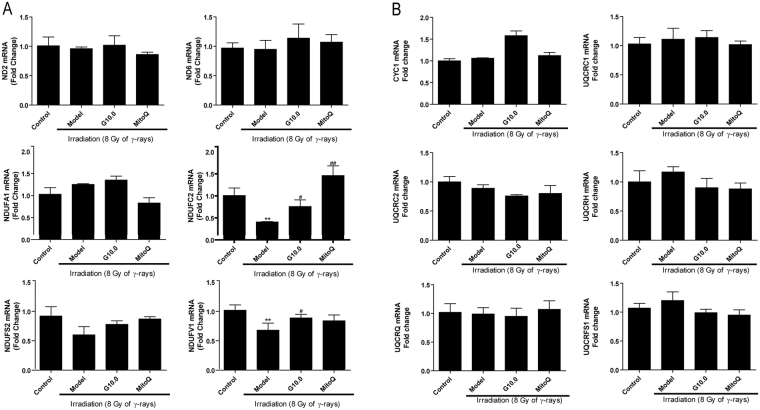
Effect of GSPs on complex I and complex III mRNA expression in irradiated HFL1 cells. mRNA expression of genes encoding proteins involved in the function of mitochondrial complex I (**A**) and complex III (**B**) was determined at 24 h after 8 Gy of irradiation. Relative mRNA levels were normalized GAPDH expression level and expressed as a fold change relative to the control group. Data are expressed as the mean ± SEM of three independent experiments. **P < 0.01 versus control group. ^#^*P* < 0.05 and ^##^*P* < 0.01 versus model group.

It is well known that ROS production occurs predominantly at mitochondrial ETC complexes I and III. We next hypothesized that the mitochondrial dysfunction and generation of ROS described above in irradiated HFL1 cells could be due to adaptations in mitochondrial respiratory ETC complexes. To investigate this hypothesis, we measured the expression of several genes encoding proteins involved in the mitochondrial respiratory chain by qRT-PCR. Figure 7A shows that the expression of NDUFC2 and NDUFV1, which encode the subunits of oxidative phosphorylation (OXPHOS) complex I, were significantly decreased in irradiated HFL1 cells (*P* < 0.05) compared with the control group. When cells were treated with GSPs (10.0 μg/ml) for 24 h before irradiation, the expression of NDUFC2 and NDUFV1 was increased. MitoQ (200 nm/L) increased NDUFV1 mRNA expression. Nevertheless, none of the genes encoding OXPHOS complex III subunits that were measured showed any change in expression (Fig. 7B). These results suggest that the inhibitory effect of GSPs on ROS production might be mediated by regulating complex I function.

### Treatment with GSPs improved the expression and enzyme activity of complex I

**Figure 8:**
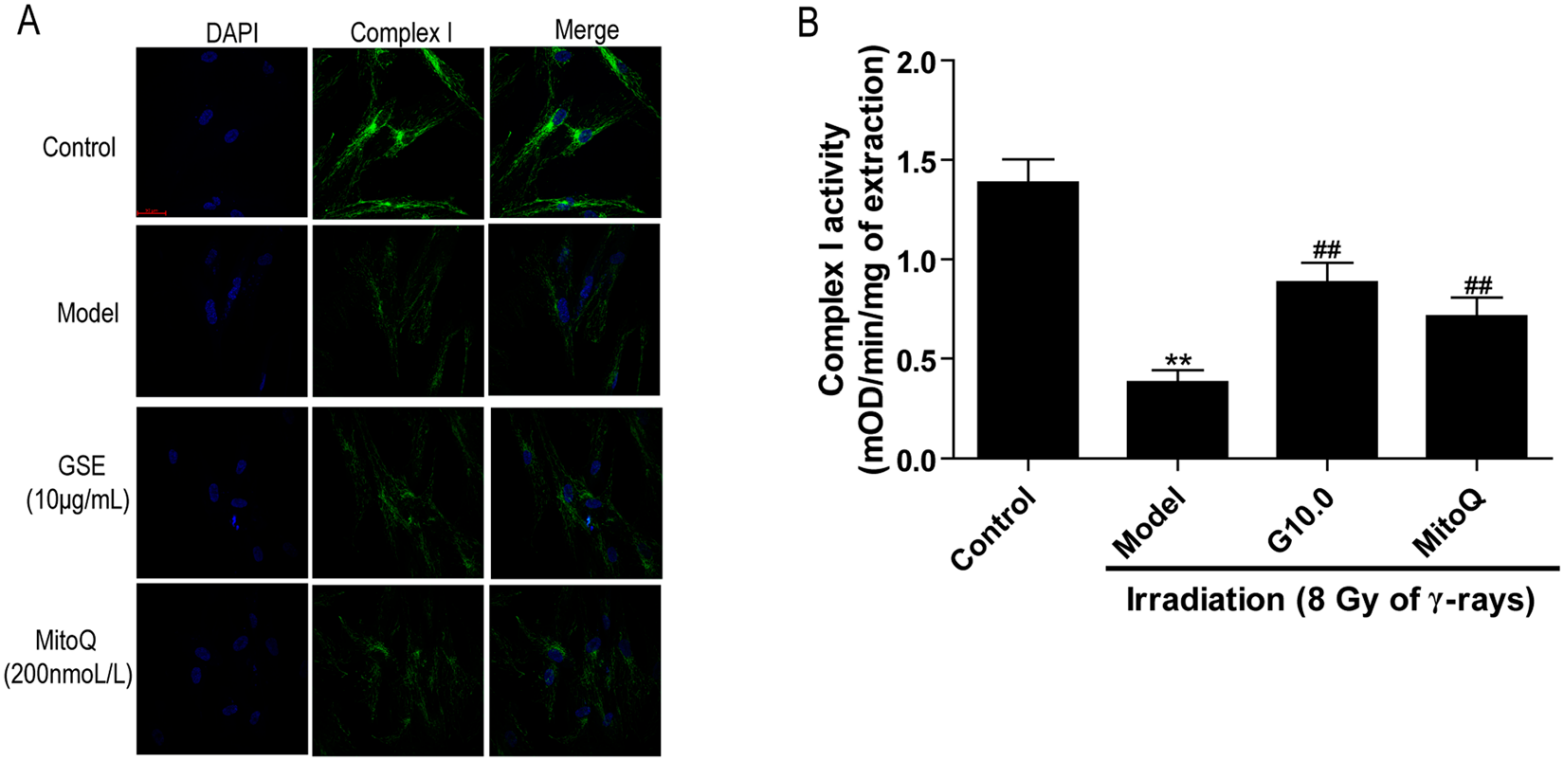
Effect of GSPs on the expression and enzyme activity of complex I in irradiated HFL1 cells. (**A**) HFL1 cells were pre-treated with GSPs (10 μg/ml), MitoQ (200 nmol/L) for 24 h before irradiation. The cells were immunostained with a complex I antibody at 72 h after irradiation. A representative confocal image from three replicates is shown. Scale bars: 20 µM. (**B**) Complex I enzyme activity was measured using spectrophotometry and expressed as ΔmOD/min/mg protein. The data were expressed as the mean ± SEM of three independent experiments. *P < 0.05 and **P < 0.01 versus control group. ^#^*P* < 0.05 and ^##^*P* < 0.01 versus model group.

Based upon the results described above, we chose to further explore the protein expression and enzyme activity of complex I in irradiated HFL1 cells. Immunofluorescence analysis confirmed that the expression of complex I was reduced 72 h after irradiation (Fig. 8A). Concurrently, the enzyme activity of complex I in irradiated HFL cells was approximately 4-fold lower than that of control cells. Nevertheless, GSPs or MitoQ increased complex I expression and enzyme activity (*P* < 0.01) (Fig. 8B) in irradiated cells.

## Discussion

To the best of our knowledge, this study is the first report regarding the inhibitory effects of GSPs on irradiation-induced cell differentiation in the human lung fibroblast cell line HFL1 *in vitro*. We clarify that irradiation leads to increases in cellular and mitochondrial ROS levels, up-regulation of mitochondria respiration and ATP production, increased proton leak, increased glycometabolism, decreased mitochondrial membrane potential, disturbance of mitochondrial fission and fusion homeostasis, impaired mitochondria complex I activity, activated p38MAPK-Akt-Nox4 pathway and lung fibroblast cell differentiation. Furthermore, we report that GSPs can significantly reduce irradiation-induced cellular and mitochondria ROS production, improve mitochondrial dysfunction and dynamics, glycometabolism and inhibit p38MAPK-Akt-Nox4 pathway in irradiated HFL1 cells.

It is well known that both radiotherapy and accidental irradiation result in fibrosis in many tissues including the lungs, liver, skin and kidneys^19^. Fibrosis is the marker of many pathological organizational reconstructions and causes clinical disease. In our previous study, radiation pneumonitis and pulmonary fibrosis occurred in the lung after irradiation in rats (data not shown), which is consistent with other reports^2,20^. During pulmonary fibrogenesis, excessive amounts of extracellular matrix components, such as α-SMA, fibronectin and collagen, are deposited and may lead to scarring and destruction of the lung architecture. Myofibroblasts are the mainly cells responsible for matrix secretion and are primarily derived from fibroblast differentiation^21^. Our present work shows that the mRNA and protein expression of α-SMA and fibronectin was significantly increased after irradiation in the human lung fibroblast cell line HFL1. In addition, irradiation significantly increased wound healing/migration of HFL1 cells (data was shown in Fig. S2A and S2B) whereas it did not affected cell proliferation, which further demonstrated that irradiation caused lung fibroblasts differentiation (Fig. S2C). Pretreatment of the cells with GSPs prevented the increase in expression of these two proteins and wound healing/migration of HFL1 cells (data was shown in Fig. S2A and S2B). GSPs also did not affect cell proliferation (Fig. S2C). These results indicate that GSPs have an inhibitory effect on irradiation-induced cell differentiation.

Previous data showed that irradiation-induced cellular ROS generation could increase profibrotic gene expression in normal human lung fibroblasts and that the cellular ROS level was associated with fibroblast differentiation^22^. In addition, it has been reported that ionizing radiation induces cellular ROS production in various cell types such as normal human lung fibroblasts, non-small cell lung cancer cells and brain microvascular endothelial cells^23–25^. Consistent with these previous reports, we found that cellular ROS were significantly increased after irradiation in HFL1 cells. Interestingly, in our work, GSPs could decrease irradiation-induced ROS production, which suggests that the inhibitory effect of GSPs on irradiation-induced fibroblast differentiation may be related to the reduced production of ROS.

More importantly, we found that mitochondrial ROS increased instantaneously in HFL1 cells after irradiation, which indicates that mitochondria may be an important source of increased cellular ROS. Blocking ROS generation in mitochondria with MitoQ, a mitochondria-targeted antioxidant, markedly reduced irradiation-induced increases in the of mRNA and protein expression of α-SMA, a well characterized marker of myofibroblast differentiation^26^. The results indicate that mitochondrial ROS are required for irradiation-induced myofibroblast differentiation. In our work, GSPs had an inhibitory effect on mitochondrial ROS production that is similar to the effect of MitoQ. Collectively, our results suggest that GSPs attenuate fibroblast differentiation, presumably by targeting mitochondria. Therefore, we focused our studies on the effect of GSPs on mitochondria.

Because it has been reported that mitochondrial ROS production is primarily related to mitochondrial ETC dysfunction^12^, we tested whether mitochondrial ETC functions, such as mitochondrial respiration and ATP content, were changed in irradiated HFL1 cells. After irradiation for 24 h, we found that irradiation promoted mitochondrial respiration with increased basal respiration and maximum respiration. In addition, ATP production was also markedly increased 1 h and 24 h after irradiation. These results are similar to the previous report in irradiated A549 cells^12^. Higher mitochondrial respiration has been linked to higher cellular energy production, so it is possible that the up-regulation of mitochondrial respiration by irradiation resulted in the up-regulation of ATP production at the early stage of irradiation. These changes in mitochondrial respiration have been identified during fibroblast differentiation recently^27^, which indicate a metabolic remodeling of the activated fibroblasts to meet the increased energetic demands associated with the enhanced secretory, synthetic and contractile function of myofibroblasts essential for fibrosis processes. In the ETC, electrons are passed through a series of proteins via oxidation-reduction reactions and the last destination for an electron along the ETC is an oxygen molecule. Under normal conditions, the oxygen is reduced to produce water; however, approximately 0.12–2% of O_2_ incorporated for respiration is estimated to be turned into ROS, and this has been particularly well documented for complex I and complex III^12^. According to these data, it is possible that the upregulated ETC functional state contributes to mitochondrial ROS production because more O_2_ is incorporated for respiration. In our present work, we found that the levels of mitochondrial respiration and ATP production did not change significantly between cells that had been pre-treated with GSPs or MitoQ and the control cells. Because the regulatory effect of GSPs on the mitochondrial ETC is similar to that of the mitochondria-targeted antioxidant MitoQ, we propose that GSPs could be used to improve the mitochondria ETC and reduce mitochondrial ROS production.

Furthermore, increased proton leak was also observed for 24 h after irradiation in our work, which demonstrates that irradiation caused abnormalities in the mitochondrial ETC. It is well documented that mitochondrial proton leak plays an important role in mitochondrial coupling efficiency and ROS production^28^. The proton leak, which includes basal leak and inducible leak, dissipates the mitochondrial membrane potential through the re-entry of protons into the mitochondrial matrix without generating ATP^28^. Basal proton leak contributes significantly to the basal metabolic rate of a resting mammal. The inducible leak can be activated by superoxide or peroxidation products and contributes to heat production. Previous studies have reported that there is a protective feedback loop between ROS and proton leak under which increased ROS production can increase mitochondrial proton leak and decreased proton leak can reduce ROS production^29,30^. Thus, we can conclude that the increased proton leak is due to an increase in ROS production in the irradiated HFL1 cells, and the reduced proton leak in GSPs-treated cells can bring about the inhibition of ROS production. However, excessive proton leak may result in inhibition of the respiratory chain complexes, which has been demonstrated in intact C2C12 myoblasts treated with a lipophilic positively charged moiety of triphenylphosphonium^31^. Therefore, a plausible explanation of our results may be that in the early stages, ROS-induced proton leak protects mitochondria from oxidative damage to some extent but subsequently a large amount of proton leak negatively affects the activity of the ETC complexes.

Given that ROS generation in the mitochondria primarily occurs at complexes I and III of the respiratory chain, we further investigated the changes in complexes I and III in irradiated HFL1 cells. Our study demonstrated down-regulation of the nuclear DNA-encoded complex I subunit genes NDUFC2 and NDUFV1, whereas the mitochondria encoded genes ND2 and ND6 showed no significant change after exposure to irradiation in human lung fibroblast cells. Additionally, complex III subunit genes showed no significant change compared to the control group. This suggests that irradiation causes complex I dysfunction and nuclear genomic instability in HFL1 cells. It has been demonstrated that NDUFV1 gene mutation leads to complex I deficiency in muscle and cultured fibroblasts from pediatric patients^32^. The NDUFC2 gene has higher mutation rates and has been recommended as a target candidate for treating colorectal carcinoma tumorigenesis^33^. The NDUFC2 gene also shows lower expression in papillary thyroid carcinoma patients^34^. Additionally, complex I deficiency is mainly caused by nuclear-encoded subunit gene mutations^35^, and our results showed that both the protein expression level and enzyme activity of complex I were decreased after irradiation. Interestingly, GSPs could elevate the mRNA expression of NDUFC2 and NDUFV1 in irradiated cells and increase complex I activity, which further indicates that the regulatory effect of GSPs on mitochondrial ETC might target complex I in the mitochondria.

Complex I, a multi-subunit enzyme, serves as the main electron entry point in the respiratory chain and is important for respiration in many aerobic organisms. Additionally, complex I has been identified as the main source of cellular ROS in a previous study^36^. Yashida *et al*. has reported that irradiation-induced decreased activity of complex I, results in the release of ROS from the mitochondrial electron transport chain (ETC), and causes persistent oxidative stress^37^. On the other side, excess mitochondrial ROS would leads to oxidative damage in the mitochondria and affects the activity of complex I and complex III^38,39^. Our study showed that the expression level and enzyme activity of complex I in the ETC were reduced in HFL1 cells 72 h after irradiation. In addition, mitochondrial ROS levels peaked at 1 h and declined at 3 h after irradiation, but at 72 h, the ROS levels of irradiated cells were still higher than the normal group, which indicates that the cells were continuing to suffer from ROS attack. Therefore, it is conceivable that irradiation damages complex I, which bring about mitochondrial dysfunction and ROS production, and then a sustained increase in mitochondrial ROS further leads to complex I deficiency. Finally excessive ROS levels induce cell differentiation.

As the balance of mitochondrial fusion and fission are involved in complex I dysfunction^40^, we further studied the changes in mitochondrial dynamics after irradiation. After irradiation, mitochondrial fragmentation was elevated in HFL1 cells. Additionally, a decrease in the mitochondrial membrane potential was observed in irradiated HFL1 cells. Increased *drp1* and decreased *mfn1/2* mRNA expression was also observed in the irradiated cells. *Drp1* is an important mitochondrial fission-related gene and *mfn1/2* is the main factor that regulates mitochondrial fusion^41^. These results indicate that irradiation leads to impaired mitochondrial morphology and an imbalance in mitochondrial dynamics in HFL1 cells. It has been reported that the disturbance in mitochondrial homeostasis results in an increase in ROS production after irradiation with ionizing radiation^42^. Furthermore, Huang *et al.* reported that aberrant mitochondrial homeostasis impairs mitochondrial complex I activity^40^. In addition, increased mitochondrial fission after ionizing radiation in normal human fibroblast-like cells involves delayed mitochondrial ROS production^41^. According to the discussion above, complex I dysfunction might be the primary source of mitochondrial ROS production. Additionally, irradiation disturbs mitochondrial homeostasis (fission and fusion), which might lead to complex I dysfunction. Our results demonstrated that GSPs could modulate mitochondrial fission and fusion and improve mitochondria morphology and mitochondrial membrane potential in irradiated HFL1 cells. Therefore, we can propose that the inhibitory effect of GSPs on mitochondria ROS production might be due to improvements in complex I activity induced by regulating mitochondria dynamics.

The above experiments suggested that irradiation increased mitochondrial respiration at the early stage in HFL1 cells, whereas it impaired mitochondrial activity with decreased Complex I activity, reduced mitochondrial potential and disrupted mitochondrial dynamics, at the later stage. In general, mitochondrial dysfunction could impair oxidative phosphorylation and the citric acid cycle, and then lead to decreased ATP production. However, ATP production still significantly increased at 72 h in irradiated HFL1 cells in our study. It is speculated that increased ATP production was mainly from enormous activation of glycolysis at the later stage of irradiation. Therefore, to explore whether the mitochondrial dysfunction leads to increased glycolysis and enormous activation of glycolysis contributed to increased ATP production, we further investigated the levels of glycolysis (indicated by lactate assay and glucose uptake assay). Our results showed that irradiation significantly increased glucose consumption and lactate release in HFL1 cells (*p* < 0.05), which indicated that irradiation increased the activation of glycolysis at the later stage and increased ATP was mainly from glycolysis. Pretreatment with GSPs or MitoQ could attenuate glucose uptake and lactic acid accumulation in irradiated cells (*p* < 0.05).

In addition, to test the changes of the plasma signaling pathway involved in fibroblast transdifferentiation in response to mitochondrial ROS, the phosphorylation levels of p38MAPK and Akt were determined. We found that phosphorylation of p38MAPK and Akt increased after irradiation, and GSPs could significantly decrease the expression of p-p38MAPK and p-Akt. We also found that irradiation increased Nox4 expression (Fig. S1A) in irradiated HFL1 cells. Treatment with siNox4 significantly decreased α-SMA and Fibronectin expression levels in irradiated cells (Fig. S1C), suggesting that Nox4 is involved in radiation-induced fibroblast differentiation, which is similar to the previous report^22^. Furthermore, GSPs could significantly reduced Nox4 expression in irradiated HFL1 cells. Additionally, after siNOX4 interference, HFL1 cells were pre-treated with GSPs (5 μg/ml) for 24 hours before irradiation. Expression of α -SMA and FN was detected by Western blotting after 72 hours. Interestingly, we found that GSPs reduced NOX4 expression (Fig. S1A). Previous data demonstrated that radiation can activate p38MAPK-Akt-Nox4 pathway directly or indirectly via ROS^22^. Therefore, GSPs might inhibit lung fbroblast cell differentiation by inhibiting p38MAPK-Akt-Nox4 pathway via decreasing ROS production.

In conclusion, our results indicate that irradiation promotes mitochondrial respiration, disrupts mitochondrial balance (fission and fusion), causes complex I dysfunction, increases glycometabolism, leads to accelerated ROS production and finally results in fibroblast differentiation by activating p38MAPK-Akt-Nox4 pathway. GSPs exhibited an inhibitory effect on cellular and mitochondrial ROS production, the regulation of ETC function, and p38MAPK-Akt-Nox4 pathway in irradiated HFL1 cells. According to these results, we conclude that the inhibitory effect of GSPs on HFL1 cell differentiation involves improved mitochondrial complex I activity induced by regulating mitochondrial homeostasis, which reduces ROS production and then lead to inhibition of p38MAPK-Akt-Nox4 pathway.

## Methods

### Cell culture and treatment

Human fetal lung fibroblasts (HFL1) were obtained from the Shanghai Cell Bank of the Chinese Academy of Sciences (Shanghai, China) and were cultured in F12K medium (Ginuo Biomedical Technology Company, Hangzhou, China) supplemented with 10% fetal bovine serum (FBS), 1% penicillin and 1% streptomycin at 37 °C in a 5% CO_2_ humidified atmosphere. The GSPs were dissolved in a small amount of dimethylsulfoxide (DMSO) and diluted with complete cell culture medium [maximum concentration of DMSO, 0.1% (v/v) in medium] before being added to sub-confluent cells (60–70% confluence). After being serum-starved in serum-free medium for 24 h, HFL1 cells were incubated for 24 h with various concentrations of GSPs or MitoQ (200 nM) in F12K medium with 10% FBS, exposed to 8 Gy γ-radiation, and then cultured for an additional 1–72 h without changing the medium. Cells treated with DMSO [0.1% (v/v)] only in F12K medium with 10% FBS for the indicated time served as the vehicle control. The model cells were also treated with vehicle (0.1% DMSO) in F12K medium with 10% FBS. The cells were used to assess the effects of GSPs and MitoQ on radiation-induced differentiation.

GSPs were received from Zhengzhou Lion Biological Technology Company (Zhengzhou, China) and contain approximately 98% proanthocyanidins. The composition of the product is 12.45% monomer, 62.15% oligomer and 25.4% polymers, which was checked by HPLC.

### Quantitative RT-PCR

The mRNA expressions of α-SMA, fibronectin, mfn1, mfn2, opa1, drp1, fis1, complex I and complex Ш were quantified by SYBR Green-based real-time PCR using specific primers (see Supplementary Table). HFL1 cells were pretreated with the desired doses of GSPs (2.5, 5.0, 10 μg/ml) or MitoQ (200 nM) in DMSO for 24 h. Then, the cells were irradiated with 8 Gy and incubated for 72 h. Total RNA from the cells was then isolated using a RNAiso Plus kit (Takara Biotechnology Co., Ltd., Dalian, Japan). The cDNA was synthesized from 1 µg of total RNA using an iScript^TM^ cDNA synthesis kit using random decamer primers (Bio-Rad, USA). The cDNA was amplified using SYBR Green mix on the Bio-Rad CFX96 Touch™ Real-Time PCR Detection System (Bio-Rad Laboratories, California, USA). Cycle threshold values were normalized for amplification of GAPDH. The relative mRNA expression levels of these target genes were assessed using the 2^−∆∆Ct^ relative quantitative method for each sample. All samples were analyzed in triplicate.

### Western blots

HFL1 cells were pretreated with the desired doses of GSPs (2.5, 5.0 or 10 μg/ml) or MitoQ (200 nM) in DMSO for 24 h. Then, the cells were irradiated with 8 Gy and incubated for 1 h or 72 h. Total cellular samples were washed twice with ice-cold PBS and incubated for 10 min in lysis buffer. Protein concentrations were determined using the BCA Protein Assay Kit (Sangon Biotech, Shanghai, China). Equal amounts of sample protein (20 μg) were separated using SDS-PAGE and transferred to polyvinylidene difluoride (PVDF) membranes. The membranes were blocked with 5% non-fat milk reagent dissolved in 1 × phosphate-buffered saline with 0.05% Tween-20 and then incubated with anti-α-SMA (Abcam, Cambridge, UK), fibronectin, p38MAPK, p-p38MAPK, Akt, p-Akt (Santa Cruz, CA) and GAPDH antibodies (Sangon Biotech, Shanghai, China) at 4 °C for 12 h. Protein bands were detected by incubating with horseradish peroxidase-conjugated secondary antibodies (Sangon Biotech, Shanghai, China), which were visualized with enhanced chemiluminescence reagent (Beyotime, Jiangsu, China).

### Immunofluorescent staining

Immunofluorescent staining of HFL1 cells was performed on microcoverslips in six-well tissue culture plates. The cells were serum-starved for 24 h and then pretreated with GSPs (10 μg/ml), MitoQ (200 nM) for 24 h. Next, the cells were irradiated with 8 Gy and incubated for 72 h. The cells were washed and fixed in 4% formaldehyde before being stained with antibodies against complex I (1:500, Abcam, Cambridge, MA, USA), α-SMA (1:100, Abcam) or fibronectin (1:200, Santa Cruz, CA, USA) overnight at 4 °C, followed by staining with secondary antibodies (fluorescein isothiocyanate-labeled goat anti-rabbit IgG; Santa Cruz) for 1 h at room temperature. The nuclei were stained using 4, 6-dia-midino-2-phenylindole (DAPI; Beyotime, Jiangsu, China).

### ROS measurements

Intracellular and mitochondrial ROS were measured using 2, 7-dichlorodihydrofluorescein diacetate (DCFH-DA; 488-nm excitation/525-nm emission; Beyotime, Jiangsu, China) and MitoSOX red (514-nm excitation/585-nm emission; Invitrogen, Carlsbad, CA), respectively. After irradiation, HFL1 cells were incubated with either DCFH-DA (10 µM) for 15 min or MitoSOX red (5 µM) for 30 min at 37 °C in the incubator. Finally, fluorescence was detected by flow cytometry. To confirm the localization of MitoSOX Red in mitochondria, the cells were also incubated with MitoTracker Green (1 μmol/L) (Invitrogen, Carlsbad, C) for 30 min at the same time. The images for both MitoSOX Red and MitoTracker Green were detected using confocal microscopy (Zeiss LSM 700; Carl Zeiss, Inc., Thornwood, Germany) and overlaid using LSM software.

### Measurement of mitochondrial morphological change

The effect of GSPs on mitochondrial morphological change in irradiation-induced HFL1 cells was assessed as previously described with slight modification^41^. HFL1 cells were seeded in confocal dishes (NEST, New Jersey, USA) and pretreated with GSPs (10 μg/ml) or MitoQ (200 nM) for 24 h. Then, the cells were irradiated with 8 Gy and incubated for 72 h. Next, the cells were washed with PBS twice and then incubated with MitoTracker Green (1 μmol/L) (Invitrogen, Carlsbad, CA) for 30 min at 37 °C. The images were detected using confocal microscopy (Zeiss LSM 700; Carl Zeiss, Inc., Thornwood, Germany) and overlaid using LSM software.

### Assessment of mitochondrial membrane potential (MMP)

HFL1 cells were pre-treated with GSP (10 μg/ml) or MitoQ (200 nmol/L) for 24 h. Then, the cells were irradiated and incubated for 72 h. MMP was estimated by staining the cells with the fluorescent dye JC-1 (Beyotime, China) according to the manufacturer’s directions. Briefly, the cells were harvested and transferred to 1.5-mL tubes, and then incubated with JC-1 (5 μg/ml) in a 37 °C non-CO_2_ incubator for 20 min after washing twice with PBS. Subsequently, the cells were collected and analyzed on a flow cytometer using 488 nm excitation with 530/30 nm and 585/42 nm bandpass emission filters. A minimum of 20,000 cells per sample was acquired and analyzed using FlowJo software.

### Assessment of cellular ATP concentration

Cellular ATP content was measured using the ATP Assay Kit (Beyotime, China) according to the manufacturer’s instructions. The luminescence of the sample was measured using an Infinite M200 Microplate Reader (Tecan, Switzerland). Protein concentration was determined using the BCA Protein Assay Kit. ATP standard curves were established, and the ATP concentrations were expressed as μmol/g of protein.

### Lactate assay

The cells were cultured in 96-well plates and pretreated with GSPs or MitoQ for 24 h and cells were irradiated and incubated for 72 h. The lactate concentration in the medium was measured with the Lactate Determination Kit (Nanjing Jiancheng, China) according to the manufacturer’s instructions.

### Glucose Uptake Assay

The cells were cultured in 96-well plates and pretreated with GSPs or MitoQ for 24 h and cells were irradiated and incubated for 72 h. The glucose concentration in the medium was determined by the glucose oxidase method. The amount of glucose consumption was calculated by subtracting the glucose concentration of cells treated with GSPs or MitoQ from the cells treated with vehicle.

### Cellular oxygen consumption

The oxygen consumption rate (OCR) in adherent HFL1 cells was measured with a XF96 Extracellular Flux Analyzer (Seahorse Bioscience, Billerica, MA, USA). The assays were initiated by replacing the growth medium from each well with 175 µl of XF Assay medium that was pre-warmed to 37 °C. The cells were incubated at 37 °C in a non-CO_2_ incubator for 60 min to allow the medium temperature and pH to reach equilibrium before the first rate measurement. After an OCR baseline measurement, 25 µl of oligomycin (OL), carbonyl cyanide 4-(trifluoromethoxy)phenylhydrazone (FCCP), rotenone and antimycin A solutions were sequentially added to each well to reach working concentrations of 1 µM, 1 µM, 1 µM and 1 µM, respectively, and changes in the OCR were analyzed.

### Assay of complex I enzyme activity

Complex I activity was measured (Complex I Enzyme Activity Microplate Assay Kit, ab109721, Abcam Mitosciences, Eugene, Oregon, USA) in HFL1 cells spectrophotometrically using cell lysates according to the manufacturer’s instructions. The activity of complex I was expressed as ΔmOD/min/mg protein.

### Statistical analysis

Data were expressed as the mean ± SEM, and *P* < 0.05 was considered statistically significant. One-way analysis of variance (ANOVA) was used to compare means between two groups. Data analysis was performed using the SPSS (V19.0, SPSS Inc., Chicago, IL, USA).

## Electronic supplementary material

Supplementary File
