# Author Correction: Grape seed proanthocyanidins prevent irradiation-induced differentiation of human lung fibroblasts by ameliorating mitochondrial dysfunction

**DOI:** 10.1038/s41598-018-22684-0

**Published:** 2018-03-07

**Authors:** XiaoHong Yang, Tao Liu, Bo Chen, Fangqin Wang, Qunfang Yang, XiaoHong Chen

**Affiliations:** Department of Pharmacology, College of Pharmacy, Third Military Medical University, Chongqing, 400038 China

Correction to: *Scientific Reports* 10.1038/s41598-017-00108-9, published online 03 March 2017

In this Article, Figure 4 is a duplication of Figure 3. The correct Figure 4 appears below as Figure 1.

**Figure 1:**
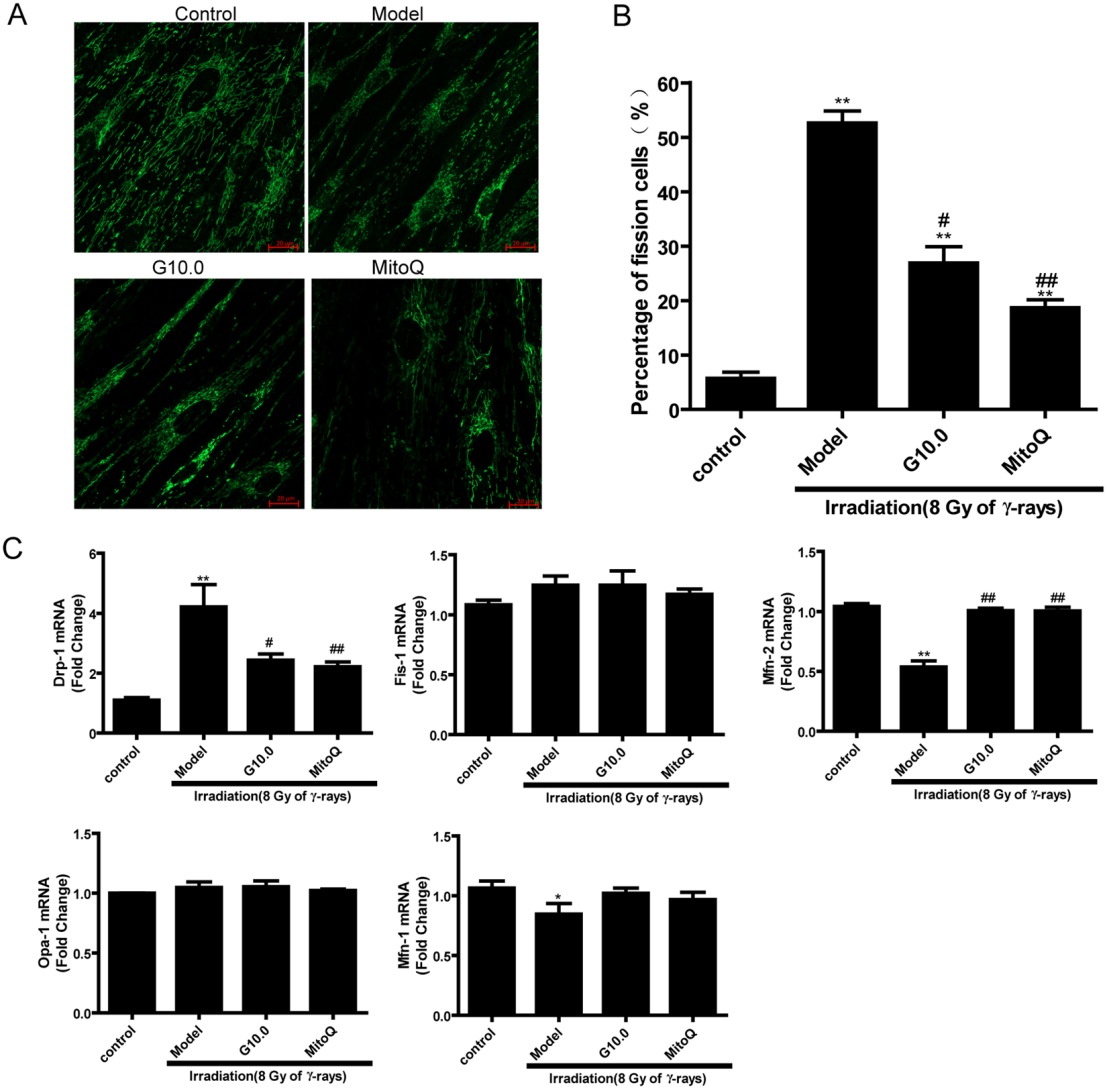
Effect of GSPs on mitochondrial dynamics in irradiated HFL1 cells. (**A**) Cells were prepared as described in the Methods and stained with MitoTracker® Green FM dye. Fluorescence images were collected by confocal microscopy. Scale bars: 20 μM. (**B**) Quantitative analysis of the percentage of fission cells is shown. (**C**) The mRNA levels of the mitochondrial fusion-related genes (mfn1, mfn2 and opa1) and fission-related genes (drp1 and fis1) were measured using qRT-PCR. Data are expressed as the mean ± SEM of three independent experiments. *P < 0.05 and **P < 0.01 versus control group. ^#^P < 0.05 and ^##^P < 0.01 versus model group.

