# Supplementary material for: Grape seed proanthocyanidins prevent irradiation-induced differentiation of human lung fibroblasts by ameliorating mitochondrial dysfunction
Source: Sci Rep. 2017 Mar 3;7:62. doi: 10.1038/s41598-017-00108-9 (PMC5427826; doi:10.1038/s41598-017-00108-9)
Supplement: Supplementary file 1 — Supplementary File [file 41598_2017_108_MOESM1_ESM.doc]

**Grape** **seed proanthocyanidins** **prevent irradiation-induced differentiation of human lung fibroblasts via ameliorating mitochondrial dysfunction**

XiaoHong Yang^1^*^+^*, Tao Liu^1^*^+^*, Bo Chen^1^, Fangqin Wang^1^, Qunfang Yang^1^, XiaoHong Chen^1^*

^1^Department of Pharmacology, College of Pharmacy, Third Military Medical University, Chongqing 400038, China.

*^+^*These authors contributed equally to this work

*****Correspondence: pharma821@163.com

[**Supplementary**](http://www.baidu.com/link?url=ZBaeYsbBTXAKE10kToaAYpc7b-MdCmZ2-LcndMZdDUfnFWF_iewWRMMUDHW6WeoS0InyR1rHFhraWCwiFyUoCG26iU4piMCLj4M-rGMITAJEnTixIe-DS9J8jeBAGbiA&wd=&eqid=a66b98a90001ce460000000355aba2a5) **table:** **primers used in this study**

| Gene | Primer | Sequence (5′-3′) | T_A_  ( ° C) | Reference | |  | |
| --- | --- | --- | --- | --- | --- | --- | --- |
| GAPDH | F | CATGAGAAGTATGACAACAGCCT | 60 | [1] | |  | |
|  | R | AGTCCTTCCACGATACCAAAGT |  |  | |  | |
| α-SMA | F | GGCGGTGCTGTCTCTCTAT | 60 | [2] | |  | |
|  | R | CCAGATCCAGACGCATGATG |  |  | |  | |
| Fibronectin | F | TCGCCATCAGTAGAAGGTAGCA | 60 | [3] | |  | |
|  |  | TGTTATACTGAACACCAGGTTGCA |  |  | |  | |
| mfn-1 | F | TTGGAGCGGAGACTTAGCAT | 51 | [4] | |  | |
|  | R | TTCGATCAAGTTCCGGATTC |  |  | |  | |
| mfn-2 | F | AGAGGCATCAGTGAGGTGCT | 56 | [4] | |  | |
|  | R | GCAGAACTTTGTCCCAGAGC |  |  | |  | |
| opa-1 | F | GGCCAGCAAGATTAGCTACG | 51 | [4] | |  | |
|  | F | ACAATGTCAGGCACAATCCA |  |  | |  | |
| drp-1 | R | AAGAACCAACCACAGGCAAC | 51 | [4] | |  | |
|  | F | GTTCACGGCATGACCTTTTT |  |  | |  | |
| fis-1 | R | CTTGCTGTGTCCAAGTCCAA | 53 | [4] | |  | |
|  | F | GCTGAAGGACGAATCTCAGG |  |  | |  | |
|  | R | TGTTATACTGAACACCAGGTTGCA |  |  | |  | |
| ND2 | F | CATATACCAAATCTCTCCCTC | 60 | [5] | |  | |
|  | R | GTGCGAGATAGTAGTAGGGTC |  |  | |  | |
| ND6 | F | GTAGGATTGGTGCTGTGG | 60 | [5] | |  | |
|  | R | GGATCCTCCCGAATCAAC |  |  | |  | |
| NDUFA1 | F | ATGTGGTTCGAGATTCTCC | 60 | [5] | |  | |
|  | R | GCAACCCTTTTTTCCTTGC |  |  | |  | |
| NDUFC2 | F | GGTTTGCATCGCCAGCTTC | 60 | [5] | |  | |
|  | R | CAGGAAAATCCTCTGGATG |  |  | |  | |
| NDUFS2 | F | ACCCAAGCAAAGAAACAGCC | 60 | [5] | |  | |
|  | R | AATGAGCTTCTCAGTGCCTC |  |  | |  | |
| NDUFV1 | F | TGAGACGGTGCTGATGGACTTC | 60 | [5] | |  | |
|  | R | AGGCGGGCGATGGCTTTC |  |  | |  | |
| CYC1 | F | TGCGGGAAGGTCTCTACTT | 60 | NM_001916.4 | |  | |
|  | R | GCACACATCCTTGGCTATCT |  |  | |  | |
| UQCRC1 | F | GAGCACCAGCAACTGTTAGA | 60 | NM_003365.2 | |  | |
|  | R | TGAAGCGGCATGGAGTAAG |  |  | |  | |
| UQCRC2 | F | GCAATTTAGGAACCACCCATTT | 60 | NM_003366.2 | |  | |
|  | R | GCCACCAACTGCTTCAATTC |  |  | |  | |
| UQCRH | F | GAGCAGTTGGAGAAATGTGTAAAG | 60 | NM_001297565.1 | |  | |
|  | R | CCGTGCAATCCTCTTCTGTAT |  |  | |  | |
| UQCRQ | F | CGCATTCGGGAGTCTTTCT | 60 | NM_014402.4 | |  | |
|  | R | CTGGATTCTTCCTCTTGGATCTC |  |  | |  | |
| UQCRFS1 | F | GTGCCTGACTTCTCTGAATACC | 60 | NM_006003.2 | |  | |
|  | R | TCCAGTTACCAAATAGGAGAAACC |  | |  | |  |
| T_A=_ annealing temperature, F= forward primer, R= reverse primer. | | | | | | | |

**
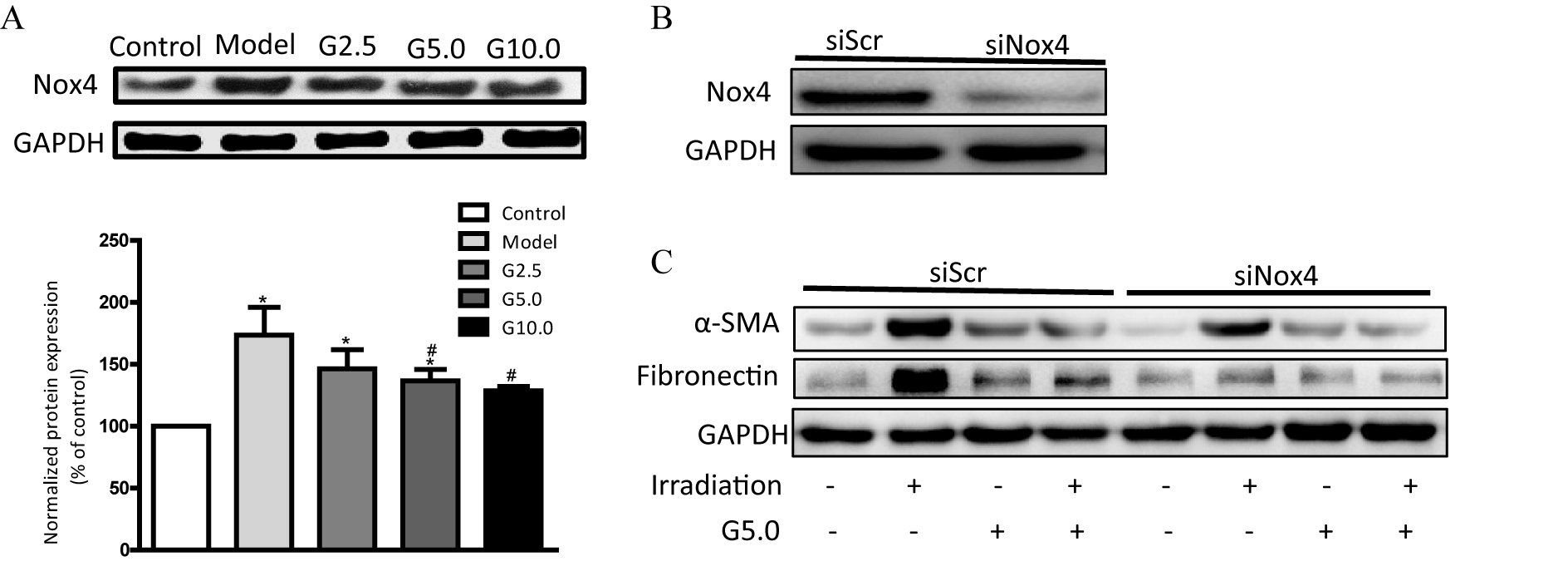
**

**Supplemental Fig. 1** The role of Nox4 in human lung fibroblast differentiation**. (A)** HFL1 cells were pre-treated with GSPs (2.5μg/ml, 5μg/ml, 10 μg/ml) for 24 hours before irradiation (γ-ray, 8Gy), and the expression of Nox4 was assessed 72 h after irradiation by Western blotting. **(B)** Six hours of transfection with 10nM siNox4 or 10nM scrambled siRNA, the levels of Nox4 was measured 72 h after irradiation by immunoblotting. **(C)** HFL1 cells were pre-treated with GSPs (5μg/ml) for 24 hours before irradiation. Expression of α -SMA and FN was detected by Western blotting after 72 hours. Protein expression was normalized to that of GAPDH. Data are representative of at least three different experiments, and the fold increase is indicated under the relevant protein bands. **P* <0.05 and ***P*<0.01 versus control group. ^#^*P* <0.05 and ^##^*P*<0.01 versus model group.

**
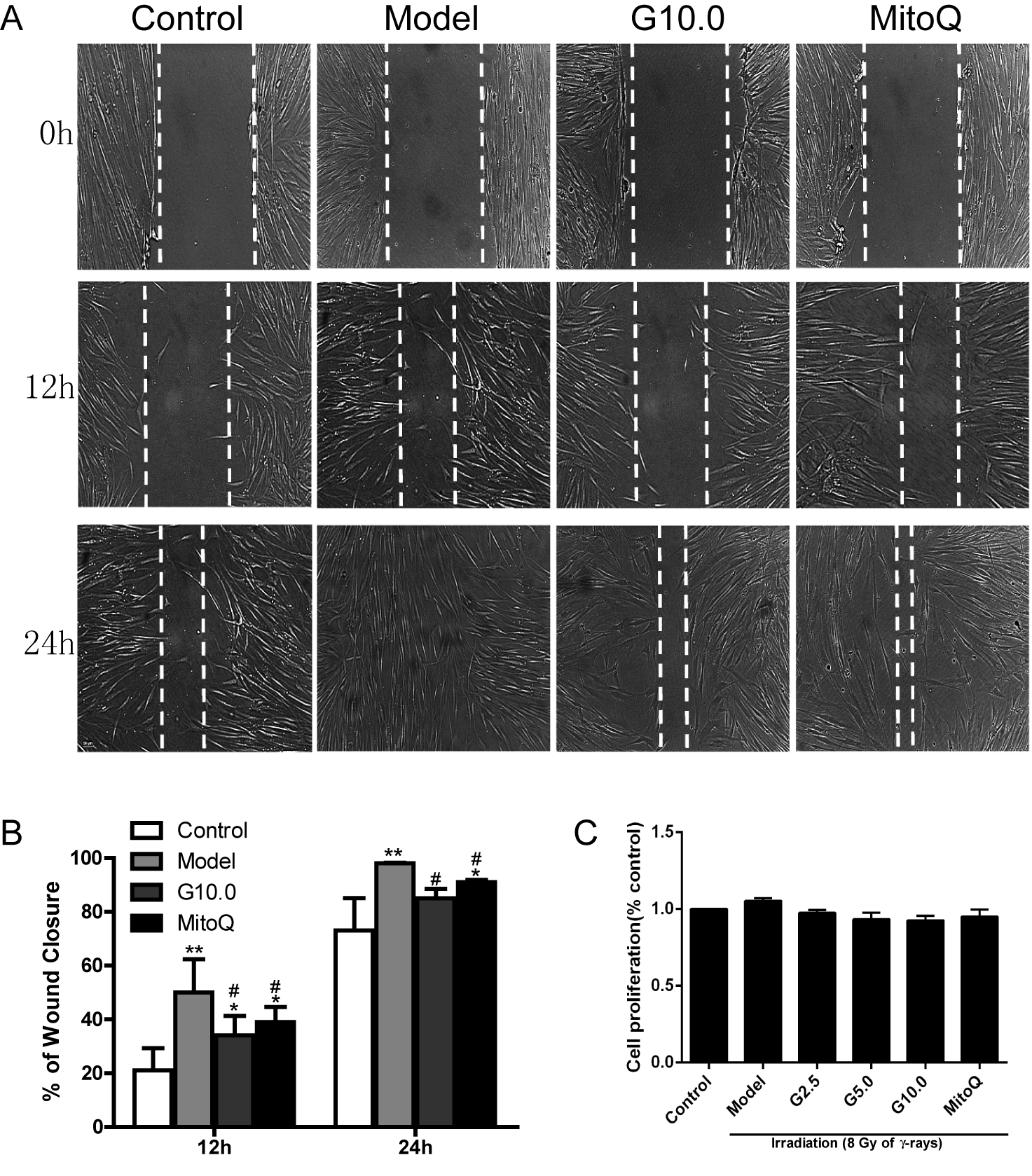
**

**Supplemental Fig. 2** **(A)** Effects of GSPs on irradiated HFL1 cells migration and proliferation. Cell migration was determined by wound-healing assay. HFL1 cells were pre-treated with GSE (10 μg/ml) or MitoQ (200nmol/L) for 24 hours before irradiation and scratched with a 200ul pipet tip to create a wound. Photographs of the wounds were recorded after 0, 12, and 24 h, at the identical location of the initial image and wound closure area was quantitated using ImageJ. The images are representative of at least three different experiments with similar results. **(B)** The relative percent of wound closure was calculated at 12h and 24 h in HFL cells before the complete would closure. **(**C) HFL cells were pre-treated with GSE (10 μg/ml) or MitoQ (200nmol/L) for 24 hours before irradiation and were further cultured for another 72 hours. Then cell proliferation was determined by LDH assay. Data represent the means and SEM of three independent experiments. **P*<0.05 and ***P*<0.01 versus control group. ^#^*P*<0.05 versus model group.

References

[1] Ko, J. C.; Tsai, M. S.; Weng, S. H.; Kuo, Y. H.; Chiu, Y. F.; Lin, Y. W. Curcumin enhances the mitomycin C-induced cytotoxicity via downregulation of MKK1/2–ERK1/2-mediated Rad51 expression in non-small cell lung cancer cells. *Toxicology and Applied Pharmacology* 255:327-338; 2011.

[2]Jain, M.; Rivera, S.; Monclus, E. A.; Synenki, L.; Zirk, A.; Eisenbart, J.; Feghali-Bostwick, C.; Mutlu, G. M.; Budinger, G. S.; Chandel, N. S. Mitochondrial reactive oxygen species regulate transforming growth factor-β signaling. *Journal of Biological Chemistry* **288:**770-777; 2013.

[3]Kawata, M.; Koinuma, D.; Ogami, T.; Umezawa, K.; Iwata, C.; Watabe, C.; Miyazono, K. TGF-β-induced epithelial-mesenchymal transition of A549 lung adenocarcinoma cells is enhanced by pro-inflammatory cytokines derived from RAW 264.7 macrophage cells. *The journal of biochemistry* 151: 205-216; 2011.

[4] Kawata, M.; Koinuma, D.; Ogami, T.; Umezawa, K.; Iwata, C.; Watabe, T.; Miyazono, K. TGF-beta-induced epithelial-mesenchymal transition of A549 lung adenocarcinoma cells is enhanced by pro-inflammatory cytokines derived from RAW 264.7 macrophage cells. Journal of biochemistry 151:205-216; 2012.

[5]Salehi, M. H.; Kamalidehghan, B.; Houshmand, M.; Yong Meng, G.; Sadeghizadeh M.; Aryani,O.; Nafissi, S. Gene Expression Profiling of Mitochondrial Oxidative Phosphorylation (OXPHOS) Complex I in Friedreich Ataxia (FRDA) Patients. *Plos one* 9: e94069; 2014.
